# An Exploratory Study of “Selfitis” and the Development of the Selfitis Behavior Scale

**DOI:** 10.1007/s11469-017-9844-x

**Published:** 2017-11-29

**Authors:** Janarthanan Balakrishnan, Mark D. Griffiths

**Affiliations:** 1 Thiagarajar School of Management, Madurai, India; 2 International Gaming Research Unit, Psychology Department, Nottingham Trent University, Nottingham, NG1 4FQ UK

**Keywords:** Selfitis, Selfie-taking, Selfitis Behavior Scale, Social media, Selfies

## Abstract

In 2014, stories appeared in national and international media claiming that the condition of “selfitis” (the obsessive taking of selfies) was to be classed as a mental disorder by the American Psychiatric Association and that the condition could be borderline, acute, or chronic. However, the stories were a hoax but this did not stop empirical research being carried out into the concept. The present study empirically explored the concept and collected data on the existence of selfitis with respect to the three alleged levels (borderline, acute, and chronic) and developed the Selfitis Behavior Scale (SBS). Initially, focus group interviews with 225 Indian university students were carried out to generate potential items for the SBS. The SBS was then validated using 400 Indian university students via exploratory factor analysis (EFA). Six factors were identified in the EFA comprising environmental enhancement, social competition, attention seeking, mood modification, self-confidence, and social conformity. The findings demonstrate that the SBS appears to be a reliable and valid instrument for assessing selfitis but that confirmatory studies are needed to validate the concept more rigorously.

On March 31, 2014, a news story appeared in the *Adobo Chronicles* website that the American Psychiatric Association (APA) had classed “selfitis” as a new mental disorder (Vincent 2014). The article claimed that selfitis was “the obsessive compulsive desire to take photos of one’s self and post them on social media as a way to make up for the lack of self-esteem and to fill a gap in intimacy” (p.1). The same article also claimed there are three levels of the disorder—borderline (“taking photos of one’s self at least three times a day but not posting them on social media”), acute (“taking photos of one’s self at least three times a day and posting each of the photos on social media”), and chronic (“uncontrollable urge to take photos of one’s self round the clock and posting the photos on social media more than six times a day”). The story was republished on numerous news sites around the world but it soon became clear the story was a hoax. However, just as empirical research into “internet addiction” started following the publication of a hoax criteria for “internet addiction disorder’ by Ivan Goldberg in 1995 (Widyanto and Griffiths 2010), it appears that the same is arguably true for “selfitis.”

Ever since Griffiths (1995) published the first paper on “technological addictions,” there has been a marked increase in research into internet addiction, online videogame addiction, mobile phone addiction, social media addiction, etc. There have also been other new technologically related mental health disorders such as “nomophobia” (no mobile phone phobia; King et al. 2010), “technoference” (constant intrusions of technology into everyday life; McDaniel 2015) and “cyberchondria” (feeling ill after searching online for the symptoms of illnesses; Lewis 2006). “Selfitis” appears to be another candidate to add to this growing list although there has been little research on its phenomenology or its sub-components. The present study empirically explored the concept and collected data on the existence of selfitis with respect to the three alleged levels (borderline, acute, and chronic) and developed a new psychometric scale to assess sub-components of selfitis.

## A Brief Overview of Selfie Behavior

According to the Oxford Dictionary, a “selfie” refers to “a self-portrait photography of oneself (or oneself with other people), taken with a camera or a camera phone held at arm’s length or pointed at a mirror, which is usually shared through social media” (Sorokowski et al. 2015). Ma et al. (2017) describe the taking of selfies in terms of self-presentation theory, which is applied to impress others. The taking of selfies is arguably not a stand-alone action because it takes on other dimensions when it is shared via social media. Such actions enable selfie-takers to present themselves in a controlled way. In recent years, selfie-taking has become an incredibly popular activity often going viral online when sharing selfies via social media domains (Frosh 2015; Rettberg 2014; Hess 2015; Roberts and Koliska 2017; Moon et al. 2016).

Research examining selfie behavior has encompassed many different areas. Researchers have investigated selfies in the context of gender and race (Albury 2015), use of selfies in a political context (Baishya 2015; Deller and Tilton 2015), military selfies (Dishy 2017), luxury selfies (Marwick 2015), and the association of selfies with personal traits (Choi et al. 2017; Qiu et al. 2015). Hess (2015) noted that selfies are used in both private and public settings, where users tend to engage in both environments. Additionally, selfie-taking is more than just the taking of a photograph and can include the editing of the color and contrast, changing backgrounds, and adding other effects, before uploading the picture onto a social media platform. These added options and the use of integrative editing has further popularized selfie-taking behavior (Fox and Rooney 2015), where users can observe their selfie creations as beautiful mirrored selves (Liubinienė and Keturakis 2014). The buying of merchandise associated with the taking of selfies (such as selfie-sticks to improve the picture range) has also grown markedly in recent years (Flaherty and Choi 2016). Selfie-sticks help photographs to appear more like regular ones taken by somebody else (Dinhopl and Gretzel 2016). Despite increasing research into selfie behavior, much of the research has been from a qualitative perspective.

The taking of selfies is a self-oriented action which allows users to establish their individuality (Ehlin 2014) and self-importance (Murray 2015). According to some studies, selfie behavior is also associated with traits such as narcissism (Buffardi and Campbell 2008). Bevan (2017) investigated the role of narcissism, considerateness, and social attraction towards selfie behavior in terms of using selfie-sticks and found that selfie-stick users were perceived as less socially attractive, moderately narcissistic, and moderately inconsiderate. Halpern et al. (2016) argued that taking selfies and narcissism are reflective actions. Although there is a strong argument that narcissism has a positive effect towards taking selfies (McCain et al. 2016), other researchers have found no relationship between selfie-taking and narcissism (Re et al. 2016). McCain et al. (2016) reported that social attractiveness was the primary motivation for posting selfies. Selfie-takers try to provide a greater appeal to others in their social media space (Re et al. 2016). Charoensukmongkol (2016) reported that attention-seeking, loneliness, and self-centered behavior had a significant relationship with selfie-liking. Although initial media reports thought that selfie-taking would be a fad, it appears that the behavior has become more endemic and is a very popular activity among adolescents and emerging adults (Albury 2015).

Anecdotally, there is evidence of excessive selfie-taking which no doubt prompted the hoax story and criteria published in the *Adobo Chronicles* (Vincent 2014). One of the reasons that so many news outlets republished the story was that the criteria used to delineate the three levels of selfitis (i.e., borderline, acute, and chronic) had good face validity. Consequently, the present paper examines these three levels empirically. More specifically, the study attempts to answer two key questions: (i) what are the sub-dimensions that aid the development of selfitis? and (ii) do the identified sub-dimensions differ across the three different levels of selfitis? It is hoped that the answer to such questions will increase understanding towards selfitis functions and the determinants of such action. The target population were Indian students because India is the country that has the most users on *Facebook* (Simon 2017). It is also worth noting that deaths sometimes occur as a result of trying to take selfies in dangerous contexts and that India accounts for more selfie deaths in the world compared to any other country with 76 deaths reported from a total of 127 worldwide (Lamba et al. 2016).

## Method

The present study used an exploratory design to investigate the proposed research questions. The findings were then used to develop a scale to assess the sub-dimensions of selfitis. The study began by using focus groups to gather an initial set of items that underlie selfitis. These initial set of items were then statistically analyzed using component analysis and a rigorous validation procedure. Although the analysis relied on non-clinical convenience samples, they were likely to represent any of the three selfitis categories (i.e., borderline, acute, and chronic).

## Focus Group Interviews

### Participants

To begin scale development, 225 students (average age = 20.93 years: SD = 4.32) from two Indian university management schools were pooled and categorized into three condition groups, borderline group (*n* = 43; 15 females, 28 males), acute group (*n* = 72; 38 females, 34 males), and chronic group (*n* = 33; 22 females, 11 males). The remaining students (*n* = 72) were not categorized into any of the groups because they did not match the threshold requirement to fall into any of the three categories.

### Procedure

Seven focus group interviews were carried out (minimum = 23 min; maximum = 46 min). The first author acted as a moderator for all the focus groups. Example questions used during the focus group interviews included the following: “What compels you to take selfies?”, “Do you feel addicted to taking selfies?”, “Do you think that someone can become addicted to taking selfies?”, etc. Through the focus group interviews, 39 statements were identified that were understood to related to selfitis motivations among the participants. Exemplar quotes of the things reported by the participants are outlined in Table 1. After the screening process and removing items that were conceptually similar, 22 statements remained that were inclusive of all three levels. The 22 statements via the focus group interviews were streamlined into items assessed using a 5-point Likert scale (5 = strongly agree and 1 = strongly disagree).

**Table 1:** Three exemplar quotes from focus group interviews that helped determine subscale categories on the Selfitis Behavior Scale

Environment enhancement	Rajesh: “When the environment is active and participatory, I forget myself and immerse myself with the environment which subsequently compels me to take a selfie either alone or as a group” Nila: “Taking selfies in a specific environment helps me to remember the moment for a long time” Harish: “Friends create moments, moments create happiness and enhances the environment and it compels me to take a selfie”
Social competition	Karthik: “Sometimes I explicitly compete with my friends to get more likes for my selfies” Lakhsmi: “I feel I am lost when my friends get more likes and comments for selfies than me” Priyanka: “I invest a lot of time enhancing my selfie photos and then I upload so that I can gain a competitive winning edge”
Attention seeking	John: “I take at least fifteen different selfies to upload just one on social media” Murthy: “I spend at least twenty minutes editing and grooming the picture before uploading it in social media” Raj: “My primary reason for taking selfies or posting them in social media is to gain attention”
Mood modification	Precilla: “I take selfies to relax and energize my mood to a positive temperament” Santhosh: “Sometimes taking selfies helps me to come out of any depressive thoughts” Vijay: “Taking selfies reduces my academic stress and gives me a different mood of relaxation”
Self-confidence	Tess: “I admire myself and gain extraordinary confidence, when I see myself in selfies” Anitha: “When people like and comment on my selfie postings, my self-confidence rises greatly” Mithun: “Taking a photo of myself and seeing a response for it in a social media domain boosts my confidence level”
Subjective conformity	Aashik: “I try to show the best of my of creativity by taking different selfies, which uplifts my social status among my friends” Kapil: “Sometimes, by trying new selfie poses, my friends accept me as a strong group member” Varsha: “I feel detached from my group if I don’t take and pose frequent selfies”

### Development of the Selfitis Behavior Scale

#### Participants

Initially, 734 students were recruited for the second phase of the study. Of these, 400 students (average age = 20.72 years: SD = 3.91) satisfied the basic condition of belonging to one of the three level categories. The socio-demographic characteristics of the sample are shown in Table 2. All 400 students actively participated in the survey.

**Table 2:** Socio-demographic characteristics of the sample (*n* = 400)

Characteristics		Frequency	%
Gender	Male	230	57.50
	Female	170	42.50
Level of selfitis	Borderline	136	34.00
	Acute	162	40.50
	Chronic	102	25.50
Age	16 to 20 years	224	56.00
	21 to 25 years	136	34.00
	26 to 30 years	27	06.75
	Above 30 years	13	03.25
Number of selfies taken per day	1 to 4 selfies	223	55.75
	5 to 8 selfies	141	35.25
	More than 8 selfies	36	09.00
Number of postings per day	None	136	34.00
	At least one time to three times	162	40.50
	More than three times	102	25.50

#### Procedure and Data Analysis

The data were collected during lecture classes from 400 respondents and responses were then entered into an Excel spreadsheet to identify any outliers. All the responses were identified to be genuine, and no one was removed from subsequent data analysis. IBM SPSS Statistics version 24 was used for the analysis. First, a dimension reduction technique (factor analysis) using principal component analysis (PCA) was carried out to identify the factors contributing to selfitis. The varimax method was used to observe the rotated factor loadings. Subsequently, multivariate analysis of variance (MANOVA) test was carried to determine whether the factors differed across the groups. The SBS is outlined in Appendix 1.

## Ethics

The study was granted approval by the first author’s university research ethics committee. All participants gave their informed consent to take part in the study.

## Results

### Exploratory Factor Analysis Results

The results of a KMO test and Barlett’s test of sphericity (Table 3) confirmed that the data were adequate for carrying the principal component analysis (Schumacker and Lomax 1998). Two statements with a communality value above 0.50 were removed from the analysis. The PCA method produced six factors with an Eigenvalues ranging from 1.05 to 6.25 (i.e., environmental enhancement, social competition, attention seeking, mood modification, self-confidence, and social conformity). The six factors explained 70.9% of the total variance. The detailed values and notes are shown in Table 3. At least three items converged in each factor, and this explained the sufficient homogeneity in the measurement (Byrne 2001). All six factors identified had a Cronbach’s alpha score of more than 0.7. The overall reliability of the scale was 0.876 and individual reliability scores of each of the six subscales are reported in Table 4.

**Table 3:** Results of Exploratory Factor Analysis on the Selfitis Behavior Scale (*n* = 400)

	Factor 1	Factor 2	Factor 3	Factor 4	Factor 5	Factor 6
1.1. Item 1	*.720*	.210	.173	.178	.139	.123
1.2. Item 7	*.755*	.100	.167	.133	.181	.094
1.3. Item 13	*.728*	.251	.093	.242	.039	.113
1.4. Item 19	*.797*	.116	.147	.097	.133	.254
2.1. Item 2	.181	*.714*	.290	.058	−.003	.230
2.2. Item 8	.134	*.780*	.156	.095	.057	.182
2.3. Item 14	.203	*.748*	.252	.089	−.060	.226
2.4. Item 20	.120	*.771*	.018	−.117	.070	−.004
3.1. Item 3	.167	.194	*.814*	.118	.004	.156
3.2. Item 9	.147	.163	*.820*	.025	.045	.167
3.3. Item 15	.179	.196	*.800*	.129	.088	.165
4.1. Item 4	.114	.008	.086	*.857*	.163	.033
4.2. Item 10	.233	.028	.075	*.755*	.180	−.082
4.3. Item 16	.163	.025	.080	*.835*	.089	.047
5.1. Item 5	.097	.001	.047	.189	*.830*	.132
5.2. Item 11	.165	.076	.063	.065	*.782*	.008
5.3. Item 17	.105	−.008	.003	.157	*.839*	−.006
6.1. Item 6	.180	.057	.188	−.053	.057	*.781*
6.2. Item 12	.142	.173	.110	.014	.094	*.776*
6.3. Item 18	.126	.258	.165	.045	−.023	*.744*
Variance (%)	31.223	13.663	7.633	6.686	6.239	5.250
Cumulative variance (%)	31.223	44.886	52.219	59.205	65.444	70.693
Eigenvalues	6.245	2.733	1.527	1.337	1.248	1.050

KMO = 0.871; Bartlett’s test of sphericity = 3472.044; Significance *p* = 0.001

Extraction method: principal component analysis; rotation method: Varimax with Kaiser Normalization

Note: The value in italics represent the highest loadings for respective factors

**Table 4:** Subscales of the Selfitis Behavior Scale and their Cronbach’s alpha scores

Items	Cronbach’s alpha
Factor 1: Environmental enhancement 1.1 Taking selfies gives me a good feeling to better enjoy my environment 1.2 I am able to express myself more in my environment through selfies 1.3 Taking selfies provides better memories about the occasion and the experience 1.4 I take selfies as trophies for future memories	0.838
Factor 2: Social competition 2.1 Sharing my selfies creates healthy competition with my friends and colleagues 2.2 Taking different selfie poses helps increase my social status 2.3 I post frequent selfies to get more ‘likes’ and comments on social media 2.4 I use photo editing tools to enhance my selfie to look better than others	0.826
Factor 3: Attention seeking 3.1 I gain enormous attention by sharing my selfies on social media 3.2 I feel more popular when I post my selfies on social media 3.3 By posting selfies, I expect my friends to appraise me	0.812
Factor 4: Mood modification 4.1 I am able to reduce my stress level by taking selfies 4.2 Taking more selfies improves my mood and makes me feel happy 4.3 Taking selfies instantly modifies my mood	0.821
Factor 5: Self-confidence 5.1 I feel confident when I take a selfie 5.2 I become more positive about myself when I take selfies 5.3 I take more selfies and look at them privately to increase my confidence	0.793
Factor 6: Subjective conformity 6.1 I gain more acceptance among my peer group when I take selfie and share it on social media 6.2 I become a strong member of my peer group through posting selfies 6.3 When I don’t take selfies, I feel detached from my peer group.	0.752

### Scale Validity

The confirmatory factor analysis showed an excellent fit of the six-factor model, χ^2^/df **=** 1.381, GFI = 0.951, AGFI = 0.934, NFI = 0.940, CFI = 0.982, RMSEA = 0.031. All items for the factors loaded significantly with standardized values more than 0.60, and this satisfied the necessary condition for content validity (Nunnally 1978). The results of the fit indices and content validity confirm the scale can be replicated or used for further research in the field. Table 5 shows the average variance extracted (AVE) of each factor, and all the values of AVE were above 0.5 which confirmed the convergent validity requirements of the scale (Fornell and Larcker 1981). The diagonal values in Table 5 represent the squared root of AVE values; in all the cases it was more than the squared correlation of the respective constructs. This confirms the discriminant validity of the construct (Sánchez-Franco and Roldán 2005).

**Table 5:** √AVE and squared inter-correlation of items on the Selfitis Behavior Scale

Constructs	AVE	1	2	3	4	5	6
Subjective conformity (1)	0.503	*0.709*					
Self-confidence (2)	0.571	0.179	0.756				
Attention seeking (3)	0.641	0.544	0.181	0.801			
Mood modification (4)	0.598	0.100	0.431	0.293	0.774		
Environmental enhancement (5)	0.572	0.527	0.391	0.525	0.480	0.756	
Social competition (6)	0.551	0.580	0.098	0.591	0.172	0.546	0.743

The diagonal values represent √AVE values

## Results of MANOVA

The results of MANOVA demonstrated that the factors differed across the three category levels of selfitis intensity (Wilks’ λ = 0.374; *f* = 41.53 (12,784); *p* < 0.001). The mean results relating to the mean difference of individual factors across intensity level (ANOVA) are shown in Table 6.Among the factors, subjective conformity was identified to differ extensively across the three intensity levels of selfitis followed by social competition and attention seeking. It was observed that all the factors significantly varied across the three categories of selfitis intensity. Table 7 shows the mean and standard deviation of the factors. Examining the mean scores shows that self-confidence and mood modification had the highest mean scores in borderline condition, subjective conformity had the highest mean score in the acute condition, and attention seeking, environmental enhancement, and social competition had the highest mean scores in the chronic condition. Table 8 shows the Scheffe’s post-hoc mean comparison of the factors across the three category levels. Among the comparisons, subjective conformity was observed to have the highest mean difference between the acute and borderline categories, and social competition was identified to have the highest mean difference between the borderline and chronic categories.

**Table 6:** Analysis of variance for the identified factors on the Selfitis Behavior Scale

Dependent Variable	Mean	*F*	Sig.	Partial Eta Squared	Observed Power
Self-confidence	3.67	17.334	.001	0.080	1.000
Attention seeking	3.49	43.619	.001	0.180	1.000
Mood modification	3.61	17.724	.001	0.082	1.000
Environmental enhancement	3.58	4.556	.011	0.022	0.773
Subjective conformity	3.04	78.112	.001	0.282	1.000
Social competition	3.64	57.956	.001	0.226	1.000

**Table 7:** Analysis of variance for the identified factors on the Selfitis Behavior Scale

	Borderline		Acute		Chronic	
Dependent variable	Mean	SD	Mean	SD	Mean	SD
Self-confidence	3.95^*^	0.526	3.54	0.802	3.48	0.751
Attention seeking	3.08	0.823	3.53	0.817	4.00^*^	0.550
Mood modification	3.89^*^	0.574	3.41	0.771	3.56	0.736
Environmental enhancement	3.50	0.690	3.52	0.724	3.76^*^	0.778
Subjective conformity	2.37	0.894	3.57^*^	0.775	3.09	0.829
Social competition	3.24	0.768	3.62	0.632	4.20^*^	0.637

**Table 8:** Scheffe’s post-hoc mean differences across the three intensity categories in the Selfitis Behavior Scale

Dependent variable	Selfitis intensity level (a)	Selfitis intensity level (b)	Mean difference (a − b)	Std. error	Sig.
Self-confidence	Borderline	Acute	.415^*^	.082	.001
		Chronic	.471^*^	.093	.001
	Acute	Borderline	−.415^*^	.082	.001
		Chronic	.056	.089	.529
	Chronic	Borderline	−.471^*^	.093	.001
		Acute	−.056	.089	.529
Attention seeking	Borderline	Acute	−.459^*^	.088	.001
		Chronic	−.927^*^	.100	.001
	Acute	Borderline	.459^*^	.088	.001
		Chronic	−.468^*^	.096	.001
	Chronic	Borderline	.927^*^	.100	.001
		Acute	.468^*^	.096	.001
Mood modification	Borderline	Acute	.481^*^	.082	.001
		chronic	.326^*^	.092	.001
	Acute	Borderline	−.481^*^	.082	.001
		Chronic	−.155	.089	.081
	chronic	Borderline	−.326^*^	.092	.001
		Acute	.155	.089	.081
Environmental enhancement	Borderline	Acute	−.019	.085	.821
		Chronic	−.262^*^	.095	.006
	Acute	Borderline	.019	.085	.821
		Chronic	−.242^*^	.092	.009
	chronic	Borderline	.262^*^	.095	.006
		Acute	.242^*^	.092	.009
Subjective conformity	borderline	Acute	−1.206^*^	.097	.001
		Chronic	−.728^*^	.109	.001
	Acute	Borderline	1.206^*^	.097	.001
		Chronic	.478^*^	.105	.001
	Chronic	Borderline	.728^*^	.109	.001
		Acute	−.478^*^	.105	.001
Social competition	Borderline	Acute	−.389^*^	.079	.001
		Chronic	−.963^*^	.089	.001
	Acute	Borderline	.389^*^	.079	.001
		Chronic	−.574^*^	.086	.001
	Chronic	Borderline	.963^*^	.089	.001
		Acute	.574^*^	.086	.001

Mean difference (a − b) denotes the mean value difference between selfitis intensity level (a) and selfitis intensity level (b) for the respective category

*Denotes values significant at 0.05 level

## Discussion

The present study explored the factors that underlie selfitis and developed a new psychometric scale—the Selfitis Behavior Scale (SBS). Using focus group interviews to generate scale components followed by statistical testing (using the dimension reduction technique), six components of selfitis were identified: environmental enhancement, social competition, attention seeking, mood modification, self-confidence, and social conformity. The MANOVA results confirmed that the six factors significantly differed across selfitis intensity level (i.e., borderline, acute, and chronic) in total as well as within the groups. The SBS appears to be a useful addition to the literature and will be helpful to future research examining the psychometric properties of selfitis. The Scheffe’s test identified that most of the high significant differences within-factor came from borderline and chronic intensity categories. This also validates that there is an appreciative deviation between the lowest and highest ranges of intensity categories. Selfitis is a new construct in which future researchers may investigate further in relation to selfitis addiction and/or compulsion. Future studies could therefore psychometrically investigate the SBS with specificity to intensity level. In the following sections, the importance of the six factors underlying selfitis is individually discussed.

### Environmental Enhancement

Previous literature has identified the environment as a contributory factor in the acquisition and development of excessive substance use and behaviors (Ajonijebu et al. 2017). Environmental enhancement by taking selfies in the present study related to feeling good, self-expression, memories, and trophies. Here, the environment is enjoyable, and the taking of selfies helps create better memories. The mean score of environmental enhancement was 3.76 (out of 5) among those with chronic selfitis. However, this factor had relatively less deviation within the three intensity categories compared to the other five factors. Findings from the focus group (see Table 1) demonstrated that selfie-takers appear to feel privileged to connect with the environment via a selfie. In fact, the participants took numerous selfies after the focus groups had finished, perhaps to provide a memory of the experience or to feel good about the research they had just been involved in.

### Social Competition

Previous literature has addressed social competition can be an important component of excessive videogaming (Hsu et al. 2009; Kuss et al. 2012; Yee 2006), gambling (Parke et al. 2004), and drug administration (Piazza and Le Moal 1998). Yee (2006) went as far as asserting that social competition is an important component for those with a gaming compulsion. In case of selfitis, the social competition factor was observed to have a high mean score of 4.2 (out of 5) in the chronic selfitis category. In fact, social competition registered high mean scores compared to the other five factors. Douglas et al. (2005) noted that social creativity may serve an intermediate tactical role in creating social competition. Those with selfitis arguably employ creative tactics that serve socially competitive needs. More importantly, social competition is a personality-based action (Sutton and Keogh 2000), and this may suggest new avenues for further research to investigate the role of social competition in selfitis across different personality types.

### Attention Seeking

Research has shown that attention seeking is a crucial component of narcissism (DeWall et al. 2011), and narcissists may engage in compensatory actions to gain attention among others (Buss and Chiodo 1991; Brown and Zeigler-Hill 2004). Social media is a well-known way to gain such attention (Lee and Ma 2012) and selfie-taking behavior is often accompanied by posting on social media outlets and is indicative of a narcissistic action. The highest mean score of attention seeking was 4.0 (out of 5) in the chronic selfitis category. Many researchers have noted factors such as self-presentation and self-admiration in the use of social media (Seidman 2013; Ryan and Xenos 2011). Literature has identified attention seeking as an important variable in social media usage (DeWall et al. 2011; Seidman 2013). Although researchers have discussed attention seeking as an important variable relevant to social media usage, the present study has opened up the possibility that attention seeking is specific to selfitis.

### Mood Modification

Griffiths (2005) refers to mood modification as a subjective experience to a particular activity that typically makes the person feel better in some way. The highest mean score of mood modification was 3.89 (out of 5) in the borderline category (i.e., it was less of a factor in acute and chronic selfitis). Similar to various addictive behaviors, mood modification among those with selfitis appears to be an important factor in reinforcing behavior in both addicts and non-addicts (Griffiths 2005). Findings from the present study suggest that selfitis could perhaps be another potentially addictive behavior where mood modification is a key factor. However, further research would be needed to investigate this. Interaction in social media via mobile digital devices appear to help many individuals overcome negative mood states. Selfie-taking is another behavior via which individuals can enhance their mood. Further research is warranted on the role of mood modification in selfitis on both the positive and negative consequences of selfie-taking behavior.

### Self-Confidence

As with mood modification, the highest mean value of self-confidence was 3.95 (out of 5) in the borderline selfitis category. Previous literature has noted that low self-confidence can lead to excessive behavior and addiction (Griffiths 2000). However, the findings here provide a new angle to explore the relationship between selfitis actions and possible addiction. Self-confidence has an internal locus which can increase people’s self-efficacy (Ajzen 2002). This is particularly appropriate in case of selfitis. Tajuddin et al. (2013) reported that taking selfies increases the perception and confidence of the takers. Technology provides the means to enhance the visual aspect of selfies via various editing applications and can take individuals closer to their ideal self via a perfect selfie. The self-admiration may lead to increased self-confidence, which may have more of a direct psychological consequence than some of the other factors. However, it may be that the increased self-confidence is only experienced online and/or for a short while offline before baseline levels of self-confidence return. Consequently, future research could explore the role of self-confidence as a part of selfitis in a more nuanced way.

### Subjective Conformity

The highest mean value of subjective conformity was 3.57 (out of 5), which was the lowest among the six factors (and highest in the acute selfitis category). Subjective conformity is an individual’s obligation to follow social conformity, which is subjective to different reference groups. Previous literature has addressed the role of social conformity and excessive behavior (Oostveen et al. 1996). Technology helps individuals to create formal and informal groups using various digital tools. Any social media platform has the means to facilitate users to create groups and propose something to follow or adhere to. Through this, individuals may attain a self and social belongingness towards the group. In this context, selfie-takers appear to follow implicit protocols to gain social acceptance. In research on behavioral excess and addiction, conformity can play an important role, because people try to extend or alter their behavior for the sake of social conformance (Berndt 1979; Cialdini and Goldstein 2004). This appears to be no different for those with selfitis. Future research should explore the importance of social and subjective conformity that may facilitate selfitis.

## Limitations, Future Research, and Conclusions

The study is not without its limitations. All the data were self-report and are subject to many well-known biases (including social desirability and memory recall). The sample was a self-selecting convenience sample of Indian students and therefore is non-representative of Indian or other populations and cultures. The vast majority of the sample (90%) was below the age of 25 years; therefore, future research should attempt to examine the selfitis across different age groups and populations using more representative samples.

Initially, the taking of selfies was considered as a fad activity, but its increasing engagement and importance given by industry and academics has established it as having a strong cultural importance—at least at the time of writing. Moreover, selfie-taking has become a major leisure activity with the help of enhanced social media functions. Improving technology along with universal connectivity via mobile devices has facilitated users to post, upload, and share their selfies via social media. Since the first paper on technological addictions (Griffiths 1995), researchers have investigated various facets of excess related to technology and its applications. As with internet addiction, the concepts of “selfitis” and “selfie addiction” started as a hoax, but recent research including the present paper has begun to empirically validate its existence.

The present research conducted focus group interviews to better understand the sub-components of selfitis. Using these data, the SBS was validated and the selfie-taking behavior was examined in relation to three intensity types (i.e., borderline, acute, and chronic conditions). The qualitative focus group data from participants strongly implied the presence of “selfie addiction” although the SBS does not specifically assess selfie addiction. Furthermore, through the analysis of the quantitative data, six factors underlying selfitis among participants were identified (i.e., self-confidence, attention seeking, mood modification, environmental enhancement, subjective conformity, and social competition). It was also demonstrated that the importance of these factors differed among those classed as borderline, acute, or chronic selfie-takers.

The present research is a novel addition to the research literature examining technology-related disorders. In addition to the psychological consequences (which may be both positive and negative), the present study provides important insights for practitioners and researchers. Although the present research is primarily exploratory in nature, the findings provide the basis for future empirical research. This study arguably validates the concept of “selfitis” and provides benchmark data for other researchers to investigate the concept more thoroughly and in different contexts. The concept of selfie-taking might evolve over time as technology advances, but the six identified factors that appear to underlie selfitis in the present study are potentially useful in understanding such human-computer interaction across mobile electronic devices. Further psychological research is needed into other factors that are likely to play a role in the acquisition, development and maintenance of selfitis including personality traits, motivations, cognition, and attitudes. Overall, the findings in the present paper demonstrate that the SBS appears to a reliable and valid instrument for assessing selfitis but that confirmatory studies are needed to validate the concept more rigorously.

## Appendix 1

### Selfitis Behavior Scale

1. Taking selfies gives me a good feeling to better enjoy my environment
2. Sharing my selfies creates healthy competition with my friends and colleagues
3. I gain enormous attention by sharing my selfies on social media
4. I am able to reduce my stress level by taking selfies
5. I feel confident when I take a selfie
6. I gain more acceptance among my peer group when I take selfie and share it on social media
7. I am able to express myself more in my environment through selfies
8. Taking different selfie poses helps increase my social status
9. I feel more popular when I post my selfies on social media
10. Taking more selfies improves my mood and makes me feel happy
11. I become more positive about myself when I take selfies
12. I become a strong member of my peer group through selfie postings
13. Taking selfies provides better memories about the occasion and the experience
14. I post frequent selfies to get more ‘likes’ and comments on social media
15. By posting selfies, I expect my friends to appraise me
16. Taking selfies instantly modifies my mood
17. I take more selfies and look at them privately to increase my confidence
18. When I don’t take selfies, I feel detached from my peer group
19. I take selfies as trophies for future memories
20. I use photo editing tools to enhance my selfie to look better than others

Scoring: Responses are rated on a 5-point Likert scale: (5 = strongly agree; 4 = Agree; 3 = Neither Agree or Disagree; 2 = Disagree; 1 = Strongly Disagree). Scores are summed. The higher the score, the greater the likelihood of selfitis

Items 1, 7, 13, and 19 relate to environmental enhancement

Items 2, 8, 14 and 20 relate to social competition

Items 3, 9, and 15 relate to attention seeking

Items 4, 10, and 16 relate to mood modification

Items 5, 11, and 17 relate to self-confidence

Items 6, 12, and 18 relate to subjective conformity
